# Efficient genome editing of genes involved in neural crest development using the CRISPR/Cas9 system in *Xenopus* embryos

**DOI:** 10.1186/s13578-016-0088-4

**Published:** 2016-03-31

**Authors:** Zhongzhen Liu, Tina Tsz Kwan Cheng, Zhaoying Shi, Ziran Liu, Yong Lei, Chengdong Wang, Weili Shi, Xiongfeng Chen, Xufeng Qi, Dongqing Cai, Bo Feng, Yi Deng, Yonglong Chen, Hui Zhao

**Affiliations:** Key Laboratory for Regenerative Medicine, Ministry of Education, School of Biomedical Sciences, Faculty of Medicine, The Chinese University of Hong Kong, Hong Kong, SAR, China; Shenzhen Key Laboratory of Cell Microenvironment, Department of Biology, South University of Science and Technology of China, Shenzhen Guangdong, 518055 China; Advanced Biomedical Computing Center, National Cancer Institute, National Institutes of Health, Frederick, MD 21702 USA; Key Laboratory for Regenerative Medicine of Ministry of Education, Ji Nan University, Guangzhou, 510632 China; KIZ-CUHK Joint Laboratory of Bioresources and Molecular Research of Common Diseases, Hong Kong, SAR, China; Department of Developmental and Regenerative Biology, College of Life Science and Technology, Ji Nan University, Guangzhou, 510632 China

**Keywords:** Genome editing, Cas9, Neural crest, Gene disruption, Segmental deletion/inversion, Multiplex deletion, *Xenopus*

## Abstract

**Background:**

The RNA guided CRISPR/Cas9 nucleases have been proven to be effective for gene disruption in various animal models including *Xenopus tropicalis*. The neural crest (NC) is a transient cell population during embryonic development and contributes to a large variety of tissues. Currently, loss-of-function studies on NC development in *X. tropicalis* are largely based on morpholino antisense oligonucleotide. It is worthwhile establishing targeted gene knockout *X. tropicails* line using CRISPR/Cas9 system to study NC development.

**Methods:**

We utilized CRISPR/Cas9 to disrupt genes that are involved in NC formation in *X. tropicalis* embryos. A single sgRNA and *Cas9* mRNA synthesized in vitro, were co-injected into *X. tropicalis* embryos at one-cell stage to induce single gene disruption. We also induced duplex mutations, large segmental deletions and inversions in *X. tropicalis* by injecting *Cas9* and a pair of sgRNAs. The specificity of CRISPR/Cas9 was assessed in *X. tropicalis* embryos and the Cas9 nickase was used to reduce the off-target cleavages. Finally, we crossed the G0 mosaic frogs with targeted mutations to wild type frogs and obtained the germline transmission.

**Results:**

Total 16 target sites in 15 genes were targeted by CRISPR/Cas9 and resulted in successful indel mutations at 14 loci with disruption efficiencies in a range from 9.3 to 57.8 %. Furthermore, we demonstrated the feasibility of generation of duplex mutations, large segmental deletions and inversions by using Cas9 and a pair of sgRNAs. We observed that CRISPR/Cas9 displays obvious off-target effects at some loci in *X. tropicalis* embryos. Such off-target cleavages was reduced by using the D10A Cas9 nickase. Finally, the Cas9 induced indel mutations were efficiently passed to G1 offspring.

**Conclusion:**

Our study proved that CRISPR/Cas9 could mediate targeted gene mutation in *X. tropicalis* with high efficiency. This study expands the application of CRISPR/Cas9 platform in *X. tropicalis* and set a basis for studying NC development using genetic approach.

**Electronic supplementary material:**

The online version of this article (doi:10.1186/s13578-016-0088-4) contains supplementary material, which is available to authorized users.

## Background

The neural crest (NC) is a unique cell population that are pluripotency and highly migratory, and can differentiate into a large variety of cell types including the peripheral nervous system and facial skeletons. During embryonic development, the NC specification, migration and differentiation are tightly regulated by a sophisticated regulatory network [1]. The NC development has been intensively studied using various animal models including the *Xenopus* embryos, however, currently loss-of-function assay for studying NC development in *Xenopus* embryos are largely based on the knockdown approach of morpholino antisense oligonucleotides. Therefore, generation of gene knockout genetic model of *Xenopus* will provide new insights on NC development.

Precise and efficient genome editing by engineered endonucleases is considered as a powerful reverse genetic approach to facilitate the study of gene functions and to establish disease models. The currently available genome editing tools of engineered endonucleases like Zinc Finger Nucleases (ZFNs) and Transcription Activator-Like Effector Nucleases (TALENs) have enabled relatively fast, specific genome targeting across human cells and various model organisms [2–9]. In addition, the newly emerged platform based on the type II clustered regularly interspaced short palindromic repeats (CRISPR) system offers a RNA-guided DNA recognition platform over the current protein based platforms of ZFNs and TALENs for genetic manipulation because of its convenience and easy access features [10–13]. This bacterial based adaptive immune system consists of two short RNAs, tracrRNA and crRNA, and a single protein Cas9. The tracrRNA and crRNA can be fused as one single guide RNA (sgRNA) for a functional CRISPR system in a previous study [12]. The commonly used Cas9 is derived from *Streptococcus pyogenes* (hereafter, Cas9 refers to the spCas9). The target sequence of Cas9 can be any 20 bp sequence followed by NGG, a conserved protospacer adjacent motif (PAM) [11, 13], which restricts the *S. pyogenes* Cas9 target space to every eight base pairs on average. By now, the CRISPR systems have been shown to disrupt specific targets effectively in various cell lines and organisms [11, 14–26]. Co-injection of more than one custom sgRNAs with *Cas9* mRNA demonstrated multiplexed gene disruption in mouse embryonic stem cells [27], zebrafish [27, 28] and *X. tropicalis* [26]. Recently, human tumor-associated chromosomal translocation was also achieved by applying Cas9 and a pair of sgRNAs targeting two chromosomes [29]. In addition to genome editing, the CRISPR/Cas9 platform with small modifications have been successfully transformed into tools to knockdown gene expression [30, 31], to create engineered transcription activator or repressor [32, 33], and more recently, to visualize repetitive elements in telomeres and coding genes in living cells [34].

As a novel tool for genome editing, the specificity of CRISPR/Cas9 received increasing attentions, as it is critically essential for future therapeutic applications and biological studies. Recent studies reported obvious off-target effects of CRISPR/Cas9 occurred in human cell lines [35–37], whereas, other studies with mouse embryos, mouse embryonic stem cell and *X. tropicalis* embryos suggested that CRISPR/Cas9 induced off-target effect was at a low rate [27, 38, 39].

D10A is a Cas9 nickase in which the 10th Asp in the RuvC catalytic domain is converted to Ala. Using D10A together with a pair of sgRNAs targeted to complementary strands of a target site could also induce indel mutations and was reported to decrease the off-target effect notably [36].

In this study, we utilized Cas9 paired with sgRNAs to disrupt genes that mainly involved in NC development in *X*. *tropicalis* embryos. We have designed 16 sgRNAs targeting 15 genes that are involved in NC specification, differentiation and migration. Among them, 14 sgRNAs are effective, showing the gene disruption rate from 9.3 to 57.8 %. We demonstrated simultaneous targeting of duplex genes in *X*. *tropicalis* embryos. We indicated that CRISPR/Cas9 system could induce segmental deletion and reversion in somatic cells. Although the Cas9 showed high specificity in most tested loci, we observed obvious off-target effects when we targeted *sox9* locus. We then used Cas9 D10A nickase and a pair of sgRNAs to reduce the off-target cleavages. Our studies shed light on the design and optimization of the CRISPR/Cas9 system for genome editing in *X. tropicalis* embryos, and set up a basis to study NC development using knockout *X. tropicalis*.

## Results

### Disruption of gene involved in neural crest development by using CRISPR/Cas9

**Fig. 1:**
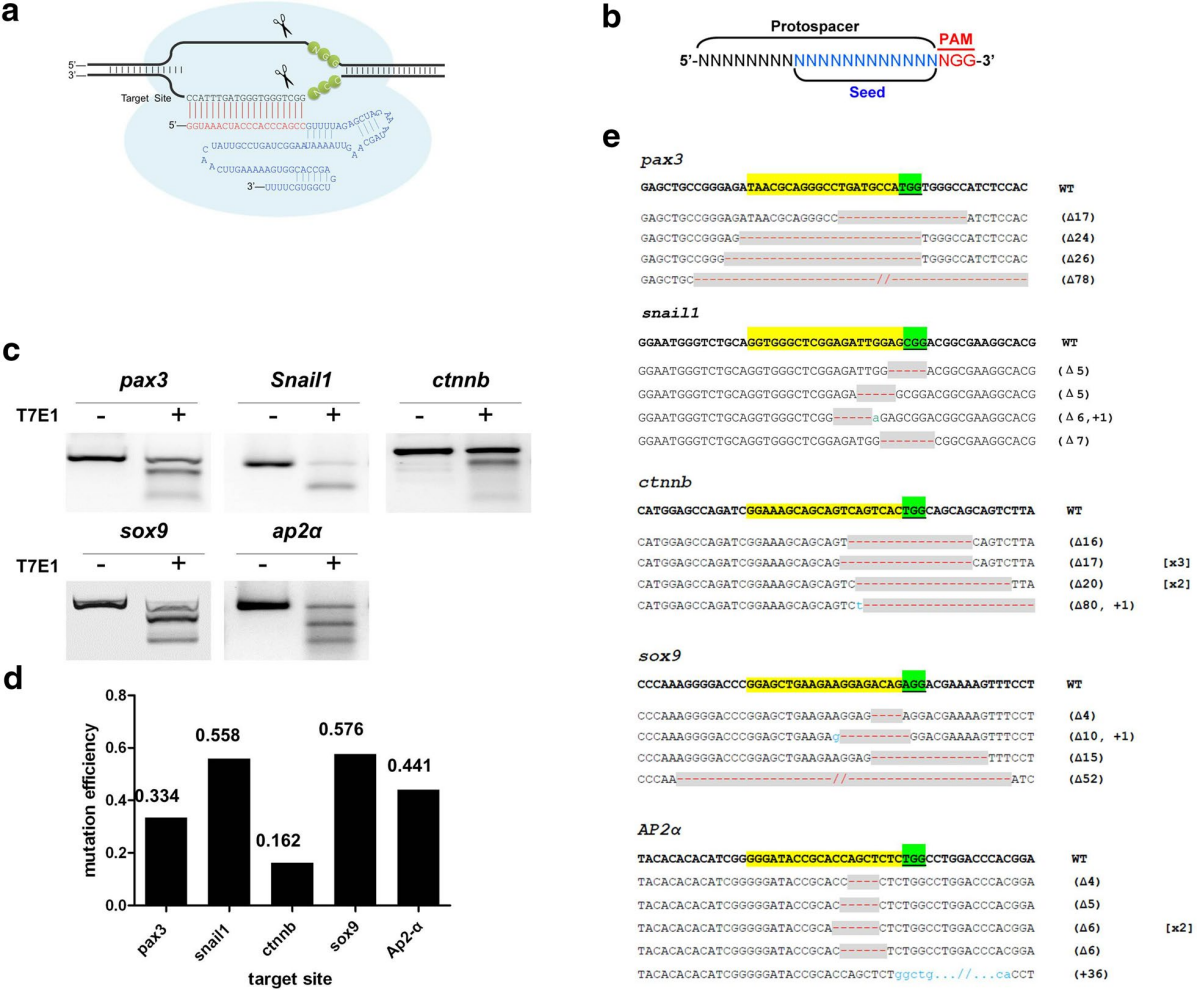
Single gene disruption targeting genes involved in NC development in *X.* *tropicalis* embryos. **a** Schematic diagram of sgRNA-guided Cas9 nuclease interacting with the DNA target site. The *red letters* indicate the target site, the *green letters* indicate the PAM and the *blue letters* indicate the sequence of tracer RNA. **b** Schematic drawing of Cas9 target site (protospacer). The protospacer adjacent motif (PAM) 3′ to the protospacer is highlighted in *red*. The *seed region* which consists of 12 nucleotides immediate 5′ to PAM is shown in *blue*. **c** T7E1 assay of somatic mutations induced by *pax3*, *snai1*, *ctnnb*, *sox9*, or *Ap2α* sgRNAs and Cas9 nuclease. **d** Somatic mutation rate calculated from T7E1 assay in (**c**). **e** DNA sequences targeted by sgRNAs at the indicated gene loci and representative somatic mutations induced in *X. tropicalis* embryos. PAM is *underlined* and highlighted in *green*. Deleted sequences are highlighted in *gray* with *red dashes* while insertions are indicated by *lowercase letters* in *blue*. The parentheses enclosed the number of deleted or inserted base pairs, while the *square brackets* show the frequencies of the mutation in the sequenced samples

The CRISPR/Cas9 has been proven to be effective for gene disruption (Fig. 1a). We therefore want to use this approach to generate genetic models that can be used for studying NC development. We designed sgRNA targeting the involved in NC development using the guideline as we previous reported [26, 40], and the schematic drawing was shown in Fig. 1b. Total 16 loci from 15 different genes were selected for targeting (Fig. 1; Additional file 1: Figure S1). We co-injected 500 pg *Cas9* mRNA and 40 pg sgRNA to obtain single gene disruption with high mutagenesis rate as we tested previously [26]. Forty-eight hours after injection, we randomly pooled five embryos injected with Cas9/sgRNA, extracted genomic DNA, amplified the targeted region, analyzed cleavage efficiency with T7E1 assay. The representative T7E1 gel images and calculated disruption efficiency were shown in Fig. 1c and d, respectively. We found that Cas9 can efficiently induce gene disruption, resulting in the cleavage efficiency from 9.3 to 57.8 %. The Cas9-induced indel mutations were confirmed by sequencing (Fig. 1e; Additional file 1: Figure S1).

### Representative phenotypes induced by CRISPR/Cas9 in G0 frogs

High disruption efficiency could imply the knockout phenotype even at G0 frog [9, 41]. Disruption of *pax3* caused the pigmentation defects at limbs revealed by lack of pigments and more transparent skin (7 out of 7), and severe defects of paralyzed upper limbs (3 in 7), which resembled some symptoms of Waardenburg syndrome in human [42] (Additional file 1: Figure S2A–D). Another example is the disruption of *tyrosinase* [43, 44]. The Tyrosinase converts tyrosine into pigment melanin, therefore disruption of *tyr* in *X. tropicalis* embryo will cause loss of pigments in larvae, which can be easily discerned. The partial albinism was also observed in adult G0 frogs (3 in 3) (Additional file 1: Figure S2E, F).

### Duplex gene disruptions induced by Cas9

**Fig. 2:**
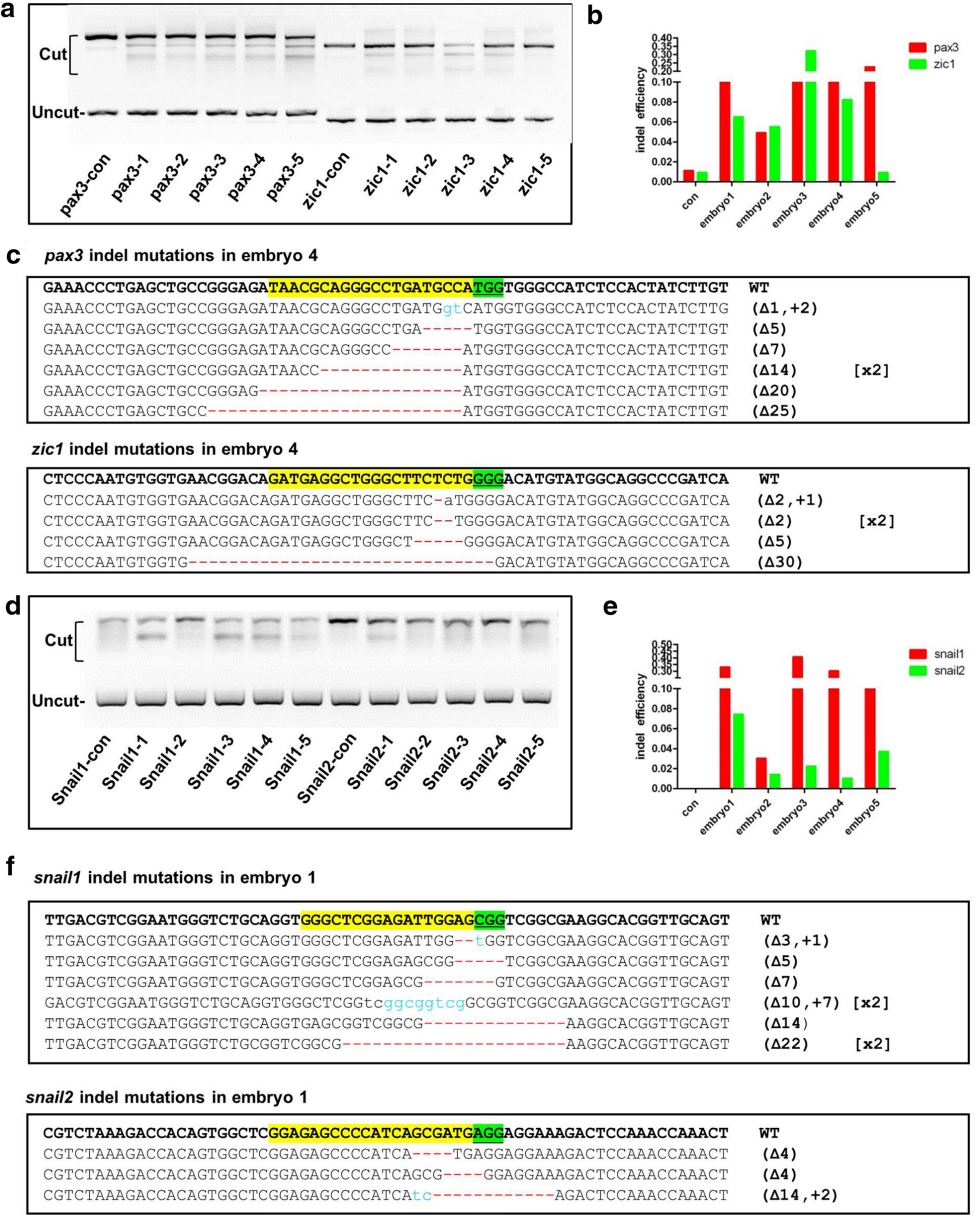
Duplex indel mutations in single embryo. **a**, **d** T7E1 assay of duplex indel mutations in the loci of *pax3* and *zic1* (**a**) or *snail1* and *snail2* (**d**) in five single embryos. **b**, **e** Quantification of the T7E1 assay in (**a**) or (**d**). **c**, **f** Representative sequencing data confirmed the indel mutations induced at the two target loci in one single embryo

A biological process usually was controlled by a group of genes. Loss-of-function may not be achieved by just disruption of a single gene as redundant members from the same gene family may exert complimentary function. Therefore multiplex gene disruption is valuable for studying the hierarchy of a gene regulatory network. During the NC specification, Pax3 and Zic1 coordinate to induce expression of NC specific transcription factors. We injected mixture of *pax3* sgRNA, *zic1* sgRNA and *Cas9* mRNA into *X. tropicalis* embryos. At 48 hpf, genomic DNA was extract from single embryo, and the locus specific PCR detected that mutations were induced at both *pax3* and *zic1* loci in 10 out of 10 embryos. T7E1 assay indicated mutation rates were 5–23 % for *pax3,* and 0.9–32 % for *zic1*. Sequencing data confirmed the somatic mutations induced by duplex sgRNAs (Fig. 2a–c).

We targeted *snail1* and *snail2* simultaneously with the same strategy. Again we found both *snail1* and *snail2* were disrupted in single embryos. Five out of five embryos harboring mutation at *snail1* and *snail2* loci, with mutation rate 3–40.5 % for *snail1* and 1–7.4 % for s*nail2* revealed by T7E1 assay. It is notable that mutagenesis rates calculated from sequencing data were higher than those obtained from T7E1 assay. Eight out of 20 and five out of 20 colonies derived DNA from embryo #4 harbor mutation in the loci *pax3* and *zic1*, respectively. Similarly, Sequencing results indicated higher mutagenesis rate for *snail2*. Three out of 19 colonies derived from embryo #2 harbored indel mutations (Fig. 2d–f).

### Targeted segmental deletion and inversions

**Fig. 3:**
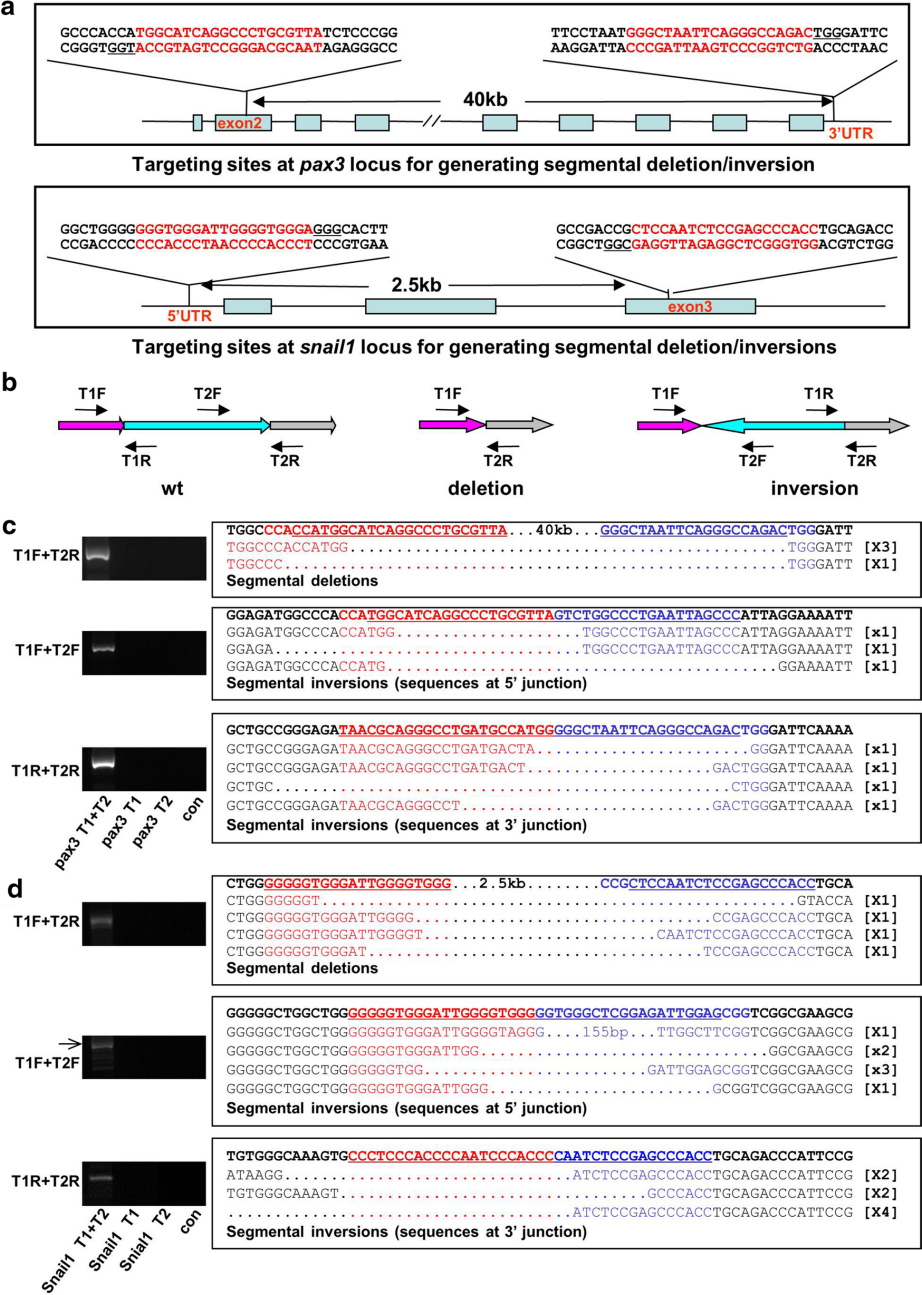
Segmental deletions and inversions induced by *Cas9* and a pair of sgRNAs in *X. tropicalis* embryos. **a** Schematic diagram showing the structure of *X. tropicalis* *pax3* and *snail1* genes, and the sgRNA targeting sequences and their locations in the two genes. **b** Schematic diagram illustrating detection of segmental deletion and inversion at targeted loci with PCR. **c**, **d** PCR assay and subsequent sequencing detected segmental deletions and inversion at *pax3* locus (**c**) and *snail1* locus (**d**). The embryos injected with Cas9 and indicated sgRNAs were collected at 2 days after fertilization. Five embryos were pooled for DNA extraction. T1, embryos injected with *Cas9* mRNA and sgRNA *T1*; T2, embryos injected with *Cas9* mRNA and sgRNA *T2*; T1 + T2, embryos injected with mixture of Cas9 mRNA, sgRNA *T1* and sgRNA *T2*. *F* forward primer; *R* reverse primer

Although CRISPR/Cas9 with a single sgRNA could induce indel-mutations with high frequency, such indel-mutations are often small (<30 bp). This particular feature cannot satisfy requirements where larger and more predictable genomic alterations are desired. Generation of large segmental deletion is very useful to study the functions of noncoding elements, such as lincRNA, miRNA clusters and *cis*-regulatory modules. It is also valuable to make animal models for the diseases caused by the segmental deletions [45] or by chromosomal inversions [46]. The ability to create large segmental deletion or inversion is therefore very useful for generating animal models or cell lines for studying human diseases. To test whether CRISPR/Cas9 can efficiently induce segmental deletion or inversion in *Xenopus* embryos, we designed another sgRNA that targeted to 3′ UTR in *pax3*, *pax3 T2* sgRNA, which is approximate 40 kb downstream of the *pax3* sgRNA (hereafter *pax3 T1* sgRNA) targeting site (Fig. 3a). We injected the mixture of *pax3T1* sgRNA, *pax3 T2* sgRNA and *Cas9* into embryos at one-cell stage. Ten injected embryos were pooled and genomic DNA was extracted at 48 hpf. The possible large segmental deletions were detected by PCRs with primers that can bridge two targeting sites (Fig. 3b). The amplicons were subcloned to TA vector, and the mutations were confirmed by sequencing (Fig. 3c). The generation of inversion induced by this pair of sgRNAs was also detected by PCR by using two forward primers from each targeting sites separately (Fig. 3b) and confirmed by afterwards sequencing (Fig. 3c). Similarly, we also created segmental deletion and inversion at *snail1* loci using a pair of sgRNAs which target *snail1* 5′ UTR and exon 3 respectively (hereafter *snail1T1* and *snail1 T2* sgRNAs). The segment between the two *snail* sgRNA targeting sites is 2.5 kb. Co-injection of *snail T1*, snail1 *T2* sgRNAs and *Cas9* also induced segmental deletion and inversion revealed by PCR assay, and then confirmed by sequencing (Fig. 3d). Thus CRISPR/Cas9 system is an effective tool to generate segmental deletions or inversions in *Xenopus* embryos, which set a basis for generating *Xenopus* models for diseases caused by segmental deletion or inversion.

### Gene disruption with sgRNA designed with 5′-NAG-3′ PAM sequence

A recent study that investigated gene regulation mediated by engineered Cas9 transcriptional activator suggested that Cas9 can also use 5′-NAG-3′ as an alternative PAM [33]. To test whether it is feasible for Cas9-mediated gene disruption in *X. tropicalis* embryos, we designed three sgRNAs targeting *id3, myosinX,* and *twist1* immediately followed by 5′- NAG-3′, respectively. We tested their efficacy in *X. tropicalis* embryos by injecting 40 pg NAG sgRNA with 500 pg *Cas9* mRNA into embryos at one-cell stage. As revealed by T7E1, these targeting induced mutagenesis efficiencies at 1.2 % for *id3*, 3.9 % for *myosin X*, and 0 % for *twist1* (Additional file 1: Figure S3). We amplified the target regions, TA cloned the amplicons, and sequenced the colonies for each of gene. None indel mutations were identified, which confirmed our observation with T7E1 assay. The low mutagenesis rates suggest that the *5′*- NAG-*3′* is not an effective PAM sequence for Cas9 in *X. tropicalis* embryos.

### Assess the potential CRISPR/Cas9 off-target effects in *Xenopus* embryos

**Fig. 4:**
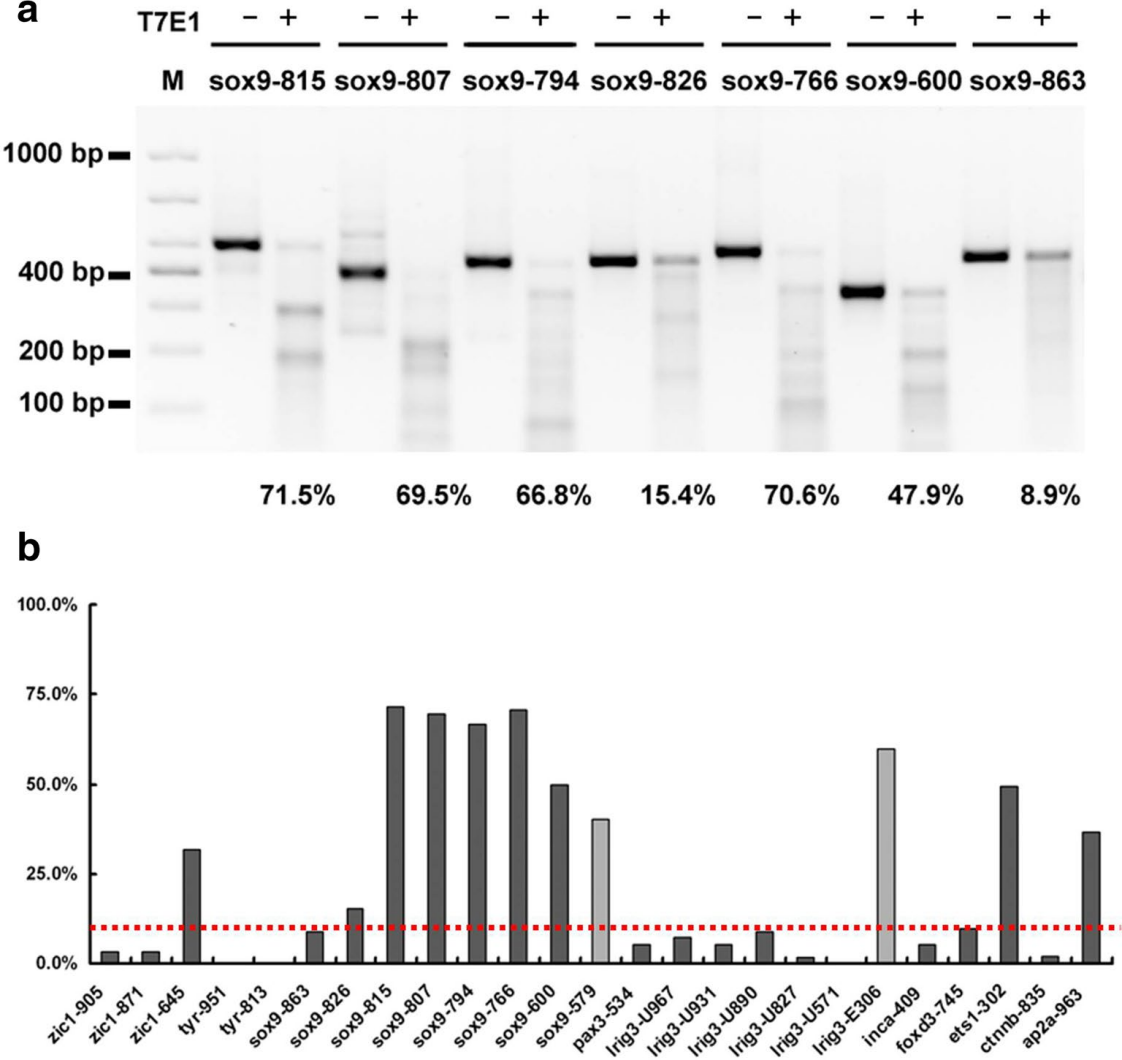
Off-target effects of CRISPR/Cas9 in *X. tropicalis* evaluated via T7E1 assay. **a** Representative gel image of T7E1 digested off-target amplicons. The percentage under *panels* indicated the frequencies of off-target effects. **b** Quantification of the cleavage frequencies of all off-target sites. No clear and specific bands after T7E1 assay were generated at *sox9*-*579* and *lrig3*-*E306* loci. The cutting efficiencies at *sox9*-*579* and *lrig3*-*E306* sites were calculated by sequencing results. *Red dash line* indicated 10 % in indels efficiency

Although CRISPR/Cas9 platform is an effective tool for genome editing, some studies with cultured cell lines indicated obvious off-targets of CRISPR/Cas9 at some loci [35–37]. However, other studies in mouse embryonic stem cells or *X. tropicalis* embryos suggested high specificities of CRISPR/Cas9 at the tested loci [38, 39]. The CRISPR/Cas9 mediated specific DNA cleavage can be significantly reduced by single mismatch at the sgRNA target sites [11, 47], especially in the seed region (12 nucleotides located in the 3′ next to PAM, Fig. 1b). We also scanned *X. tropicalis* genome to identify potential off-target sites for the 16 targeted loci, and found the potential off-target sites corresponding to 11 target loci (Additional file 1: Table S3). These potential off-target loci from the targeted embryos were amplified by locus specific PCR, and the amplicons were analyzed by T7E1 assay. Totally, 25 potential off-target loci corresponding to the 11 target loci were analyzed by T7E1 assay, and 11 off-target loci showed T7E1 positive (Fig. 4). We selected the loci with over 10 % mutagenesis rate, and subcloned the amplicons into PMD-18T vectors. Five to eleven bacterial colonies for each off-target loci were randomly picked for sequencing. Two potential off-target sites, *sox9*-*579* and *lrig3*-*E306* were failed in T7E1 analysis, since no clear and specific bands after T7E1 assay were detected due to repetitive sequences in the region. Although T7E1 assay suggested obvious off-target mutagenesis at *Ap2α*-*963* (50 %), *sox9*-*826* (15.4 %), and *zic1*-*645* (32 %) loci, sequencing data did not reveal any mutations at these loci (Additional file 1: Figure S4). However, off-target mutagenesis induced at other examined loci was profound. Among them, mutations were found in all the sequenced samples at five examined loci (*sox9*-*600*, *sox9*-*766,sox9*-*794*, *sox9*-*807*, and *sox9*-*815*). The sequence variations occurred at *ets1*-*302* were proven as nucleotide polymorphism because the uninjected embryos also harbors the same DNA alternations (Additional file 1: Figure S4). Collectively, these results indicated that CRISPR/Cas9 could induce obvious mutagenesis at some off-target loci in *X. tropicalis* embryos.

### D10A nickase reduced off-target cleavage in *Xenopus* embryos

Recent reports indicated the off-target mutations induced by Cas9 in cultured cell lines [11, 35–37]. We also observed substantial off-target effects when we used Cas9 to target *sox9* locus. Since the high specificity is essential for genome editing, we next sought to find approaches to minimize off-target effect of CRISPR/Cas9. The two nuclease domains in Cas9, HNH and RuvC, cleave the DNA strand complementary and noncomplementary to the sgRNA, respectively. Point mutation of D10A in RuvC or H840A in HNH converted Cas9 into DNA nickases [11, 12]. Recent studies with cultured mammalian cells indicated that off-target effects can be significantly reduced by using D10A nickase with a pair of sgRNAs targeting both strands [36]. We therefore test the efficiency of this approach in *X. tropicalis* embryos at *pax3* and *ets1* loci. First, we tested whether the D10A approach can induce gene disruption in *X. tropicalis* embryos. We designed a sgRNA against *pax3* at the 5′ upstream of the previous target site (*pax3 T3* sgRNA). The offset of the sgRNA pair (*pax3 T1* and *pax3 T3)* is +5 bp, while the 5′ overhang is 42 bp (Additional file 1: Figure S5A). We injected *D10A Cas9* mRNA together with *pax3* sgRNA pairs into the *X. tropicalis* embryos at stage one and detected the mutation efficiency by direct sequencing (Additional file 1: Figure S5B). The mutation efficiency induced by nickase was comparable to wild type Cas9 (36.8 vs 47.1 %). Similarly, we designed another sgRNA targeting *ets1 (ets1 T2)*. The offset of sgRNA pairs is +64 bp. Co-injection of *Cas9* mRNA and the pair of *ets1* sgRNAs induced indel mutations as well with ratio of 5.3 % (Additional file 1: Figure S5C, D).

**Fig. 5:**
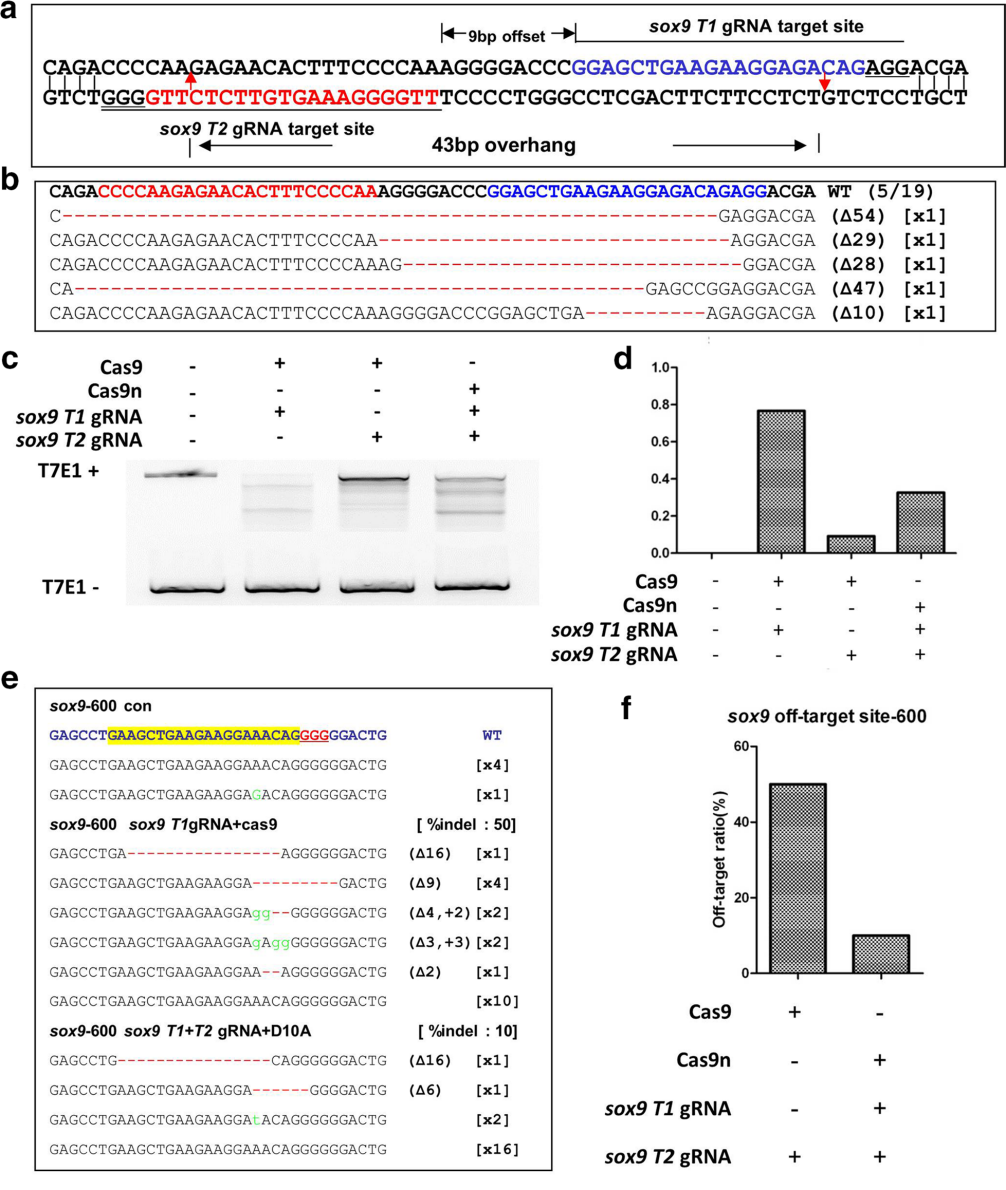
Double-nicking induced efficient genome editing with lower off-target effects. **a** Two sgRNA targeting sites in *sox9*. Letters in *blue* is the target site 1 while letters in *red* is the target site 2. The *red arrows* point the predicted cleavage sites, and the PAMs are *underlined*. **b** Sequencing data of the indels induced by D10A and the sgRNA pairs. Letters in *bold* is the wild type sequence, *red dashes* indicate deletions. **c** T7E1 assay of genome editing induce by wide-type Cas9 or by D10A. **d** Quantification of the T7E1 assay shown in (**c**). **e** Mutagenesis at a representative off-target locus (sox9-600) induced by *Cas9* and *sox9 T1gRNA* or by *D10A* and *sox9 T1 and T2* sgRNAs. PCR was performed to amplify the targeted sox9-600 locus. Amplicons harboring targeted gene fragments were TA-cloned into pMD-18T. The indel mutagenesis rates were calculated by direct sequencing. (**f**) Comparison of the off-target efficiency induced by D10A with double nicking versus wide type Cas9 with *sox9* sgRNA

The *sox9* sgRNA/Cas9 can induced indel mutation at *sox9* locus as well as the off-target sites at *sox9*-*600*, *sox9*-*766*, *sox9*-*794*, *sox9*-*807*, and *sox9*-*815* (Fig. 4; Additional file 1: Figure S4). We designed another sgRNA targeting *sox9* locus at 9 bp 5′ upstream to the *sox9* sgRNA targeting site (*sox9 T2*), both of which will generate 43 bp 5′ overhang (Fig. 5a).

We injected *D10A* mRNA together with *sox9T1* and *sox9 T2* sgRNAs into *X. tropicalis* embryos, and examined the on-target indel mutation by T7E1 and direct sequencing. The indel mutation ratio is 32.6 % (T7E1 data not shown), which is lower than we obtained with wild type *Cas9*/*sox9 T1* sgRNA (Fig. 5b). We next examined the effects of this configuration on the off-target sites. Revealed by direct sequencing, the ratio of mutation at *sox9*-*600* locus was reduced from 50 to 10 %, suggesting D10A nickase can reduce the off-target efficiency in *X. tropicalis* (Fig. 5c–f).

### Cas9-induced mutagenesis is highly heritable

The Cas9 targeted embryos were raised to sexual maturity, and crossed with wild type frogs. We collected embryos, extracted DNA from single embryos and used T7E1 assay to determine whether the embryos harbored mutations. T7E1 assay indicated that one embryo from one founder male frog of *zic1*, and three embryos from two founder male frogs of *pax3*, *snail1* and *id3*, respectively, carried the induced mutations. Such mutations were further confirmed by sequencing. So the rate of germ line transmission of the mutations ranged from 5 to 22 % (Additional file 1: Figure S5). The data again indicate that sgRNA/Cas9-induced mutagenesis is highly heritable. We are in the progress of establishing the knockout lines using the approach we described previously [48].

## Discussion

In this study, we utilized CRISPR/Cas9 platform to disrupt genes involved in NC development. In addition to the single gene disruption, we also successfully generated duplex and segmental gene disruptions. After evaluation of off-target effects in *X. tropicalis* embryos, we found that the Cas9 D10A nickase approach can reduce off-target cleavage. Our studies add versatility to this powerful tool for genome editing in *X. tropicalis* embryos. With improving the specificity, this simple and highly versatile tool will greatly expand our capacity on biological researches.

Currently, most laboratories utilize Cas9 from *S. pyogenes* for genome editing, and select 5′-N_20_-NGG-3′ sequence as its targeting sites (Fig. 1b). Based on this finding, the chance for designing sgRNA is 1/8 in a given genome. We have successfully targeted 14 genes that involved in NC formation, migration and differentiation using this approach, and will continue to establish knock-out *X. tropicalis* lines step by step. We observed some phenotypes from G0 frogs and showed the representatives in Additional file 1: Figure S2. It should be mentioned that the G0 frogs are mosaic, and G0 phenotypes could lead to adverse effects to their progeny as well. The G0 frog phenotype may provide some hints for the gene function. The gene knockout phenotype can only be determined after the knockout line is established, and we should know and distinguish these two kinds of phenotypes.

In addition, recent report suggested that 5′-NAG-3′ can also serve as PAM for Cas9. We tested three targeted sites that were followed by NAG at *twist1*, *myosin X* and *id3* loci, respectively, none of them can be disrupted (Additional file 1: Figure S3). Our data suggested that NAG cannot serve as an effective PAM for Cas9, at least in *X. tropicalis* embryos.

Multiplex mutations at different loci can be simultaneously induced in one embryo by co-injection of *Cas9* mRNA and a combination of corresponding sgRNAs [26]. The NC specification was regulated by combined effects of *pax3* and *zic1* [49]. The two transcription factors of *snail1* and *snail2* play important roles in epithelial-mesenchymal transition of NC development [50, 51]. We used Cas9 induced duplex somatic mutations at *pax3* and *zic1* loci, and *snail1* and *snail2* loci in *X. tropicalis* embryos (Fig. 2). Generation of large segmental deletion is very useful for studying the function of non-coding DNA sequences or regulatory DNA elements. It can be also used for deletion of a particular protein domain to study gene functions. Our data indicated that CRIPSR/Cas9 is efficient to generate large segmental deletion/inversions. Such abilities make CRISPR/Cas9 system a very useful platform for functional studies and for generation of diseases models.

In our pilot gene disruption studies with CRISPR/Cas9, we did not identified obvious off-target effects at some gene loci [26]. However, with increasing number of gene loci tested by CRISPR/Cas9, we found off-target in some gene loci (Fig. 4 and Additional file 1: Figure S4). Most off-target loci we identified are located in non-coding region and even intergenic region, which may not affect normal development even indel mutations were induced in these regions. We suggest 10 % T7E1 rate as a cut-off for further evaluation of the off-target cleavages. The off-target cleavages with low mutagenesis rate could be diluted through backcross with wild type animals.

The paired D10A approach can be a good solution to minimize the potential off-target effects induced by Cas9. In line with previous research work, we also observed the off-cleavages were reduced about five times when we targeted *sox9* locus with D10A and a pair of sgRNAs (*sox9T1* + *T2* sgRNAs) compared to those induced by wild type Cas9 and *sox9* sgRNA (Fig. 5). The design of sgRNA pairs followed the principles described previously. Briefly, the offsets between the sgRNA pairs are less than 100 bp, and ideally from −4 to 20 bp; and the sgRNA pair should generate a 5′ overhang other than 3′ overhang. The sgRNA pairs fulfill the above conditions may generate indel mutants.

Our criteria for identification of potential off-target sites seem reliable. A previous report indicated that even two mismatches to sgRNA were well tolerated by Cas9 to induce DNA cleavages at some loci, while three or more mismatches can significantly reduce the mutagenesis capacity [35]. Our criteria allow three mismatches, which could cover most of the potential off-target sites.

A few strategies may reduce the potential off-target cleavage induced by Cas9. (1) Use Cas9 D10A nickase coupled with a pair of sgRNAs to reduce potential off-targets. (2) Reduce injection doses of *Cas9* mRNA and sgRNA which may decrease the possible mis-binding of sgRNA to the off-target sites, or if possible, select another sgRNA binding site. A study suggests that the off-target effects occur as sequence dependent [52]. (3) Perform BLAST search, and avoid homologous sequences to reduce the possible off-target binding of gRNAs [53]. (4) Utilize the dCas9-FokI approach that was reported to reduce the off-target cleavage [54].

Taken together, we used CRISPR/Cas9 to induce indel mutations at gene loci involved in NC development in *X. tropicalis*, and set a basis for studying this developmental process using genetic animal models. When compared with the other two genome editing tools, ZFN and TALEN, the CRISPR/Cas9 platform is simpler and more convenient. Modified CRISPR/Cas9 platform with high specificity will have great impacts on biomedical research and treating human diseases.

## Conclusion

The NC cells are highly pluripotent that can differentiate into a large variety of cell derivatives. Many congenital birth defects are due to abnormalities of NC development. While *Xenopus* has been utilized as an ideal model to study the NC development, currently loss-of-function assay in *Xenopus* embryos are largely based on the MOs knockdown approach, which is hurdled by its transient effects. In this study, we took advantages of CRISPR/Cas9 system to induce targeted gene mutations in *X. tropicalis* embryos. We designed sgRNAs targeting 16 loci in 15 genes, 14 of which could induce indel mutations with relatively high efficiency. We generated duplex mutations and large fragment deletions or inversions using this system as well. The off-target effects of this system in *X. tropicalis* embryos was carefully evaluated, and we showed evidence that the Cas9 D10A nickase approach can reduce off-target cleavage.

Our studies proved CRISPR/Cas9 system as a powerful genome editing tool in *X. tropicalis* embryos. By crossing with the wild type frogs, we obtained the G1 heterozygotes harboring mutations. We can utilize this platform to study the functions of the genes involved in NC development in future.

## Methods

All experimental protocols were approved by The Government of the Hong Kong Special Administrative Region, Department of Health. The methods were carried out in accordance with the approved guidelines.

### *X. tropicalis* maintenance and husbandry

The *X. tropicalis* was purchased from Nasco Co. (USA), and the frog maintenance in principle followed the Harland *X. tropicalis* Website (http://tropicalis.berkeley.edu/home/). Synchronized embryos were obtained by in vitro fertilization using method describe previously [9, 48].

### DNA constructs

The human codon-usage optimized *Cas9* constructs were created by Church’s lab and purchased from Addgene (Cat. No. 41815). pCS2-Cas9-NLS was generated by inserting *Cas9* ORF coding sequence and relative SV40 T-antigen nuclear location signal (NLS) into *pCS2* + vector. The pCS2-Cas9-2NLS was constructed by inserting two NLS together with Cas9 coding sequence [26].

The sgRNA sequences were designed by using web-based program ZiFiT Targeter Version 4.2 (http://zifit.partners.org/ZiFiT/). Two oligonucleotides with the targeting sequence containing BsaI cutting sites at 5′ end were annealed first, and the annealed oligonucleotides were cloned into sgRNA vector pDR274 (Addgene #42250) [55] after BsaI digestion. The oligo sequences for sgRNA constructs were shown in Additional file 1: Table S1.

### Preparation of *Cas9* mRNA and sgRNA for microinjection

The pCS2-Cas9-NLS and pCS2-Cas9-2NLS were linearized by NotI (NEB), respectively. *Cas9* mRNAs were transcribed with mMESSAGE mMACHINE SP6 Kit (Life Technologies, Ambion) using linearized *Cas9* constructs as template. The *Cas9* mRNAs were purified using RNA purification kit (QIAgen) and eluted in RNase-free water. The sgRNAs were transcribed with T7 RNA polymerase after linearization of sgRNA constructs by DraI, and purified with mirVana miRNA Isolation kit (Life Technologies, Ambion).

### Microinjection

The mixture of *Cas9* mRNA and sgRNAs as indicated in the figures were injected into *X. tropicalis* embryos at one-cell stage. Microinjection was followed as previous studies [9, 48]. The injected embryos were collected for DNA extraction at 48 h after fertilization.

### T7 endonuclease 1 (T7E1) assay

T7E1 assay was basically performed as previously described [26, 56]. Briefly, 150–200 ng amplicons were denatured and re-annealed in 19 μl digestion solution using the following program: 95 °C for 5 min, 95–85 °C at −2 °C/s, 85–25 °C at −0.05 °C/s, and then maintained at 4 °C. The annealed DNA samples were digested with T7E1 (NEB M0302L), and then separated by 1.5 % agarose gel. DNA was visualized by GelRed staining. DNA signals were quantified using ImageJ software (http://rsb.info.nih.gov/ij/). The PCR primers for T7E1 assay to detect on-target cleavages were shown in Additional file 1: Table S2.

### Determination of mutagenesis rate

We normally utilize T7E1 assay to determine the mutagenesis rate. However, T7E1 can give false positive at some gene loci due to the repetitive segments or nucleotide polymorphism in *X. tropicalis* genome. We used direct sequencing to determine the mutagenesis rate (colonies harboring mutations/total colonies) in case the T7E1 assay seems not reliable.

### Identification of potential off-target sites in *X. tropicalis*

Potential off-target sites were identified in *X. tropicalis* genome based on the following criteria, (1) tolerate up to three mismatches in the sgRNA-DNA interface sequence (protospacer); (2) only allow one mismatch in the seed region (12 nucleotides 5′ to the PAM); (3) PAM is the 5′-NGG-3′ motif and the N represents four nucleotides. The potential off-target sites were amplified by PCR using genomic DNA extracted from Cas9/sgRNA injected embryos. Amplicons were either digested by T7E1 assay or subcloned into TA cloning vector for sequencing. The off-target cleavages were confirmed by DNA sequencing. The PCR primers for T7E1 assay to detect off-target cleavages were shown in Additional file 1: Table S4.

## Additional file

10.1186/s13578-016-0088-4 Supplementary figures and legends.
