# Supplementary material for: Efficient genome editing of genes involved in neural crest development using the CRISPR/Cas9 system in Xenopus embryos
Source: Cell Biosci. 2016 Mar 31;6:22. doi: 10.1186/s13578-016-0088-4 (PMC4818404; doi:10.1186/s13578-016-0088-4)
Supplement: Supplementary file 1 — 10.1186/s13578-016-0088-4 Supplementary figures and legends. [file 13578_2016_88_MOESM1_ESM.pdf]

Supplementary materials

Efficient genome editing of genes involved in neural crest development using the CRISPR/Cas9 system in *Xenopus* embryos

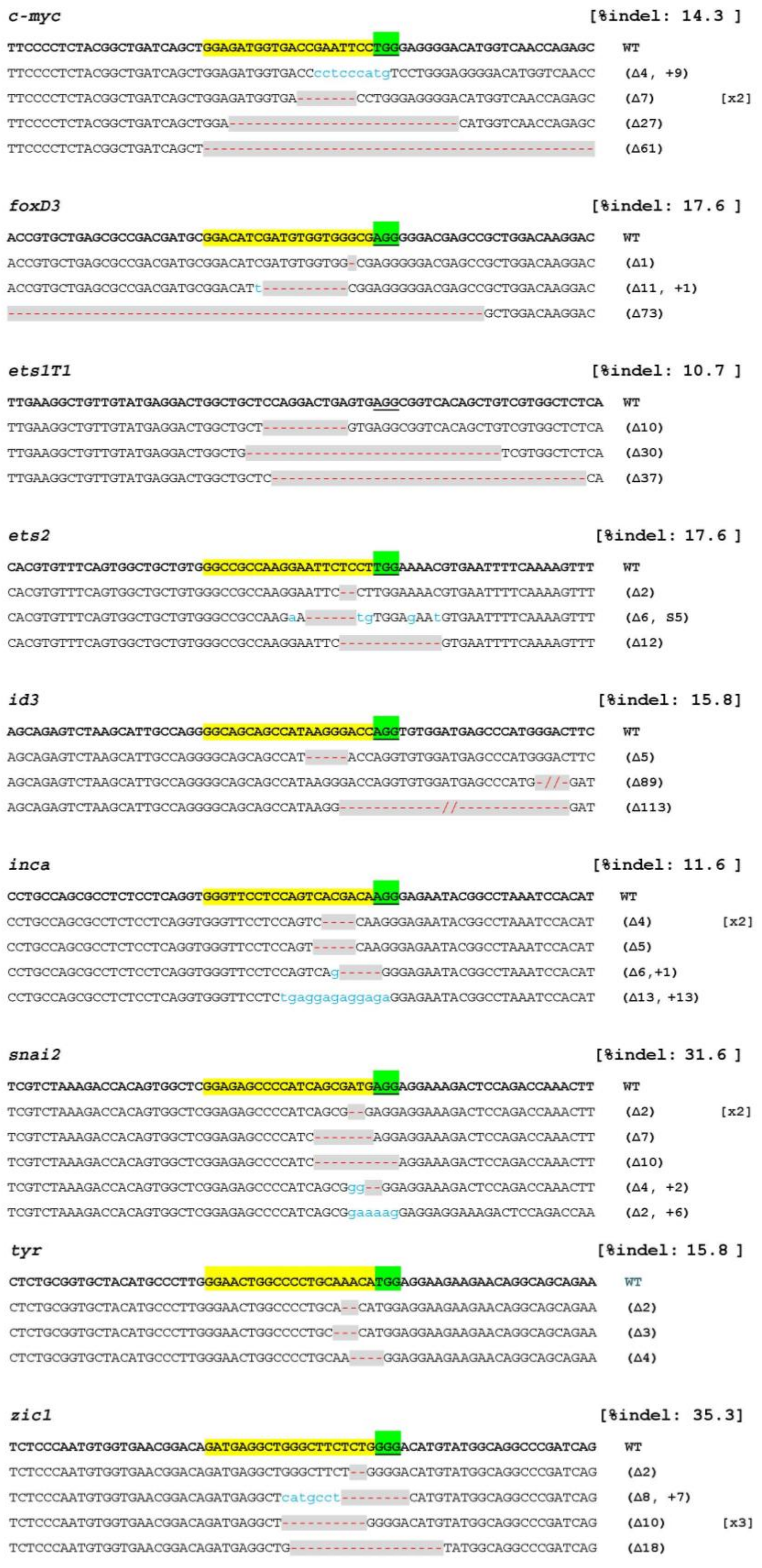

Supplementary Fig.1

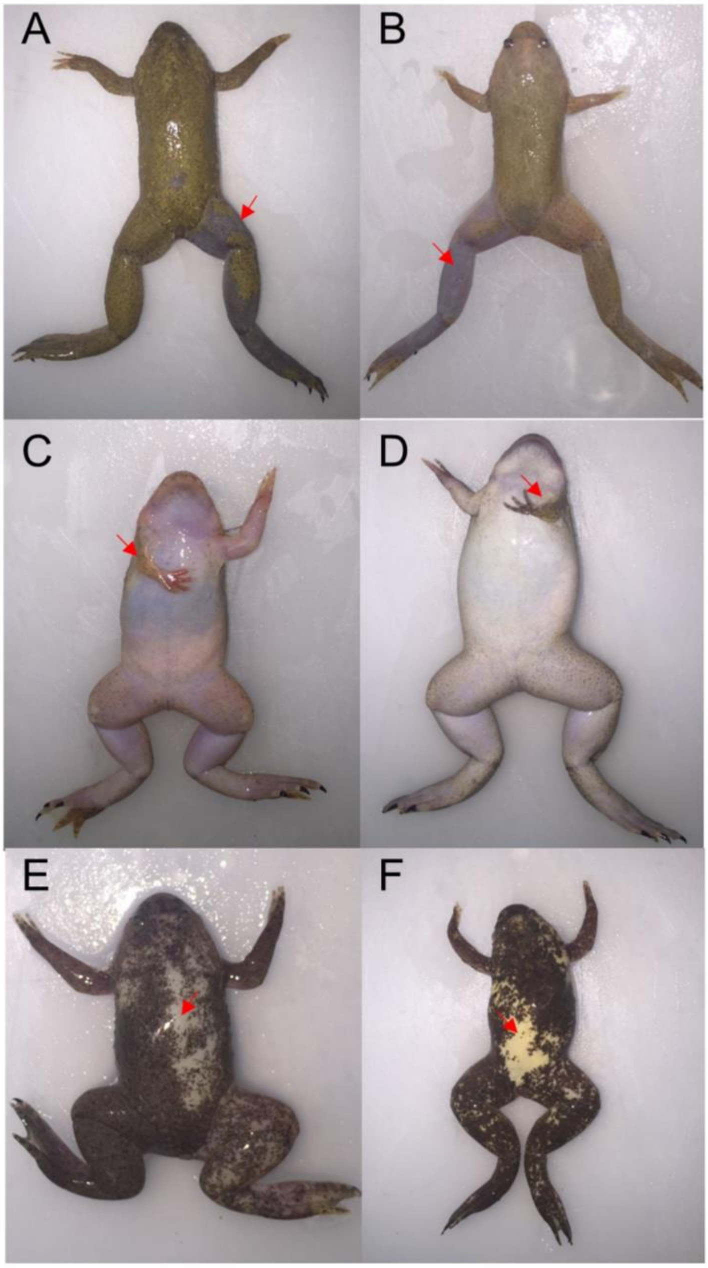

Supplementary Fig. 2

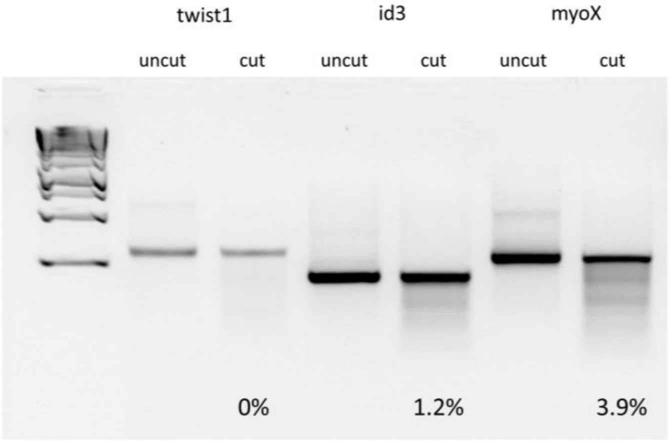


```
MyoX                                              [%indel:3.9]
AATCATCTTGTAAGGGTTGACAGATGCCAGGATGGAGCCAATATATGTCTG
AATCATCTTGTAAGGGTTGACAGATGCCAGGATGGAGCCAATATATGTCTG          [x9]
```

Supplementary Fig. 3

```
AP2α-963    (0/10)
AGTATAGGATCTGCTGGATAGCGCACCAGGTCTCAGGGTCCCACTAAGGGT     WT
AGTATAGGATCTGCTGGATAGCGCACCAGGTCTCAGGGTCCCACTAAGGGT             [×10]

zic1-645    (0/11)
CCCCACCCTTCATACATGGGTCTGGGCTTCTCTGTGGTGCCTGCACGAGGT     WT
CCCCACCCTTCATACATGGGTCTGGGCTTCTCTGTGGTGCCTGCACGAGGT             [×11]

ets1-302    uninjected
CCATCCCTTTCAATGGCATCTCCAGGGCTGAGTGAGGGTGCACTCACACTC
CCATCCCTTTCAATGGCATCTCCAGGGCTG---GAGGGTGCACTCACACTC     (-3)
CCATCCCTTTCAATGGCATCTCCAGGGCTGgAGTcGAGGGTGCACTCACAa     (+3)     [×2]
CCATCCCTTTCAATGGCATCTCCAGGaCTaGAGTcGAGGGTGCACTCACAa     (+3)

ets1-302    (5/5)
CCATCCCTTTCAATGGCATCTCCAGGGCTGAGTGAGGGTGCACTCACACTC     WT
CCATCCCTTTCAATGGCATCTCCAGGGCTG---GAGGGTGCACTCACACTC     (-3)     [×2]
CCATCCCTTTCAATGGCATCTCCAGGGCTGgAGTcGAGGGTGCACTCACAC     (+2)     [×2]
CCATCCCTTTCAATGGCATCTCCAGGaCTaGAGTcGAGGGTGCACTCACAC     (-1,+3)

lrig3-E306    (3/5)
TTGTTTGGGGAAAATAAAGAGGATTCATGGAAATGGGCATTTAAAAAAATGA    WT
GAGGATTCATGGAaatgggca..//..ttcatggaAATGGGCATTTAAAAA     (+135)   [×2]
GAGGATTCATGGAaatgggca..//..ttcatggaAATGGGCATTTAAAAA     (+134)
TTGTTTGGGGAAAATAAAGAGGATTCATGGAAATGGGCATTTAAAAAAATGA             [×2]

sox9-826    (11/11)
AAAAACATGGATCTGGAACTGAATAAGGAGACAGGGGTGTCCCCTCTAACC     WT
AAAAACATGGATCTGG-------------------------------AACC     (-31)
AAAAACATGGTtaG-------------------GGGGTGTCCCCTCTAACC     (-21,+2)
AAAAACATGGATCTGGAACTGAATAAGG----AGGGGTGTCCCCTCTAACC     (-4)
AAAAACATGGATCTGGAACTGAATAAGGAGA----GGTGTCCCCTCTAACC     (-4)
AAAAACATGGATCTGGAACTGAATAAGGAGAtAGGGGTGTCCCCTCTAACC     (-1,+1)  [×7]

sox9-579    (2/5)
GCAACAGTGGAGAGAGAGGTGAAAAAGGAGACAGAGGCGCAAAAGGAGAAC     WT
GCAACAGTGGAGAGAGAGGTGAAAAAGGAGA-----aCGCAAAAGGAGAAC     (-6,+1)  [×2]
GCAACAGTGGAGAGAGAGGTGAAAAAGGAGACAGAGGCGCAAAAGGAGAAC              [×3]
```

sox9-600 (5/5)

```
AAACCGCAGAGCCTGAAGCTGAAGAAGGAAACAGGGGGGACTGGTTCTCCG      WT
AAACCGAAATGCAT----------//-----------GCCAGCAGTCTCCG     (-66)
AAACCGTGCTAGCA--------//--------AGGGGGGACTGGTTCTCCG     (-34)
AAACCGCAGAGCCTGAAGCTGAAGAA-----------GGACTGGTTCTCCG     (-11)
AAACCGCAGAGCCTGAAGCTGAAGAAGGA---------GACTGGTTCTCCG     (-9)
AAACCGCAGAGCCTGAAGCTGAAGAAGGA-------GGGACTGGTTCTCCG     (-7)
```

sox9-766 (4/4)

```
AAACCGCAGAGCCTGAAGCTGAAGAAGGATACAGGGGGGACTGGTTCTCCG      WT
AAACCGCAGAGCCTGAAGCTGAAGAAGGA---------GACTGGTTCTCCG     (-9)     [×2]
AAACCGCAGAGCCTGAAGCTGAAGAAGGA------GGGGACTGGTTCTCCG     (-6)
AAACCGCAGAGCCTGAAGCTGAAGAAGGAgACAGGGGGGACTGGTTCTCCG     (-1,+1)
```

sox9-794 (4/4)

```
AAACTGCAGAGCCTGAAGCTGAAGAAGGAGACAGGGGGGACTGGTTCTCCG      WT
AAACTGCAGAGCCTGAAGCTGAAG---------GGGGGGACTGGTTCTCCG     (-9)
AAACTGCAGAGCCTGAAGCTGAAGAAGGAG-CAGGGGGGACTGGTTCTCCG     (-1)
AAACTGCAGAGCCTGAAGCTGAAGAAGGAtACAGGGGGGACTGGTTCTCCG     (-1,+1) [×2]
```

sox9-807 (5/5)

```
ACGTTTTTTTTGCAGCAGCTAAAGAAGGAGACAGAGGCAGCTTGCTGCAGA      WT
ACGTTTTTTTT----------------------GAGGCAGCTTGCTGCAGA     (-22)
ACGTTTTTTTTGCAGCAGCTAAAaAAaaAaAaA---//---AGAGTGCAGA     (-20,+5)
ACGTTTTTTTTGCAGCAGCTAAAGAAGG----gGAGGCAGCTTGCTGCAGA     (-5,+1)
ACGTTTTTTTTGCAGCAGCTAAAGAAGG----AGAGGCAGCTTGCTGCAGA     (-4)
ACGTTTTTTTTGCAGCAGCTAAAGAAGGAGAaCAGAGGCgGCTTGCTGCAGA    (-1,+2)
```

sox9-815 (5/5)

```
GGGGCCTCTACTGGGGAGGTGAAGAAGGAGACAGGGGCCATTGGGGGGGTG      WT
GGGGCCTCTACTG--------------------GGGGCCATTGGGGGGGTG     (-19)
GGGGCCTCaACTGGtaAGGTtAAGAAGG-GACcctGGGCCtTaGGGGGGTG     (-10,+9)
GGGGCCTCTAgTGttaAGGTTAAGAAGGAGtCAGGGGCCATTGGGGGGGTG     (-6,+6)
GGGGCCTCTACTGGGGAGGTGAAGAAGGA-----GGGCCagtcgATTGGGG     (-5,+5)
GGGGCCTCTACTGGGGAGGTtAAGAAGGAGAtgCAttGGGgggatGCCAGG     (-1,+10)
```

Supplementary Fig. 4

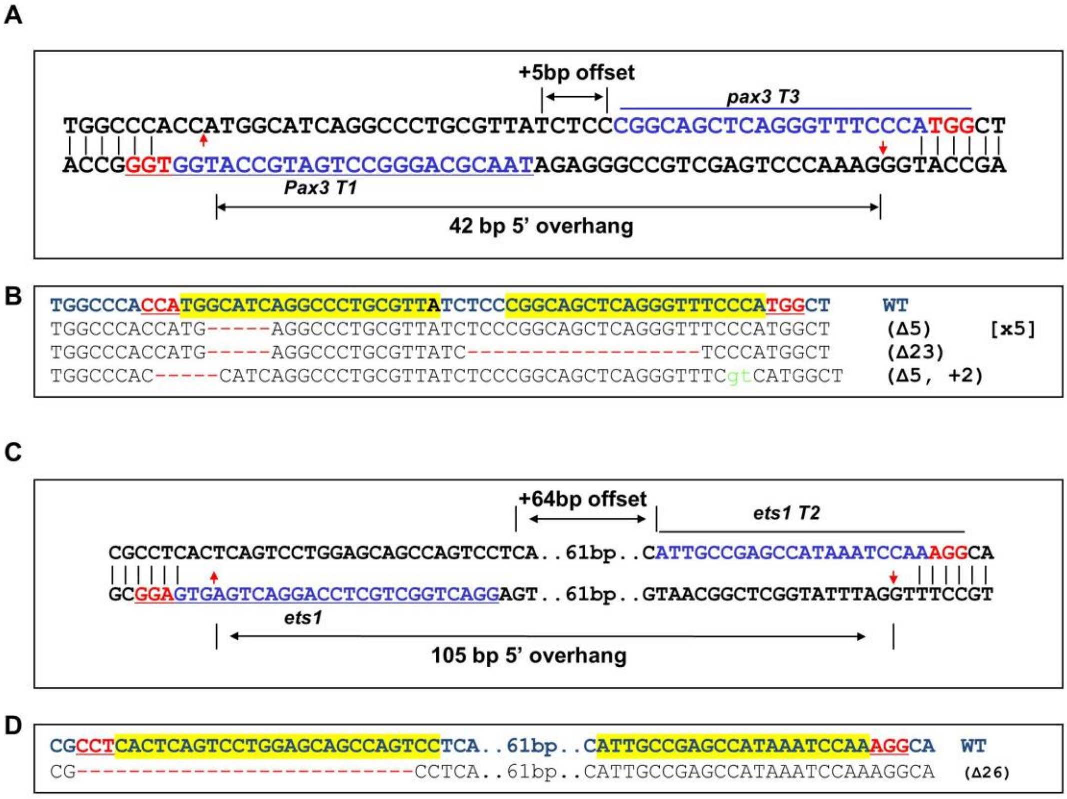

Supplementary Fig. 5

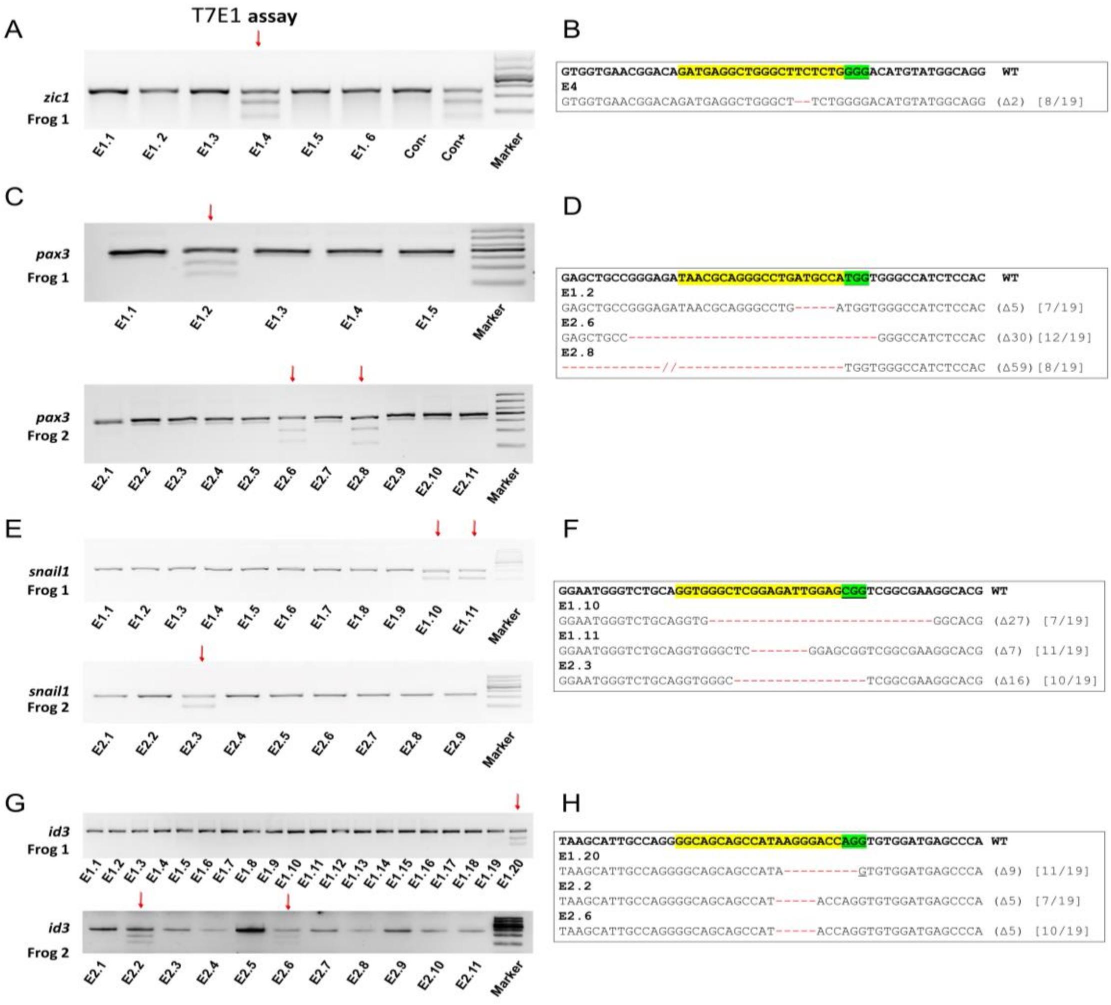

Supplementary Fig. 6

Gene	Target sequence(5'-3')	PAM	Oligonucleotide 1(5'-3')	Oligonucleotide 2(5'-3')
pax3	TAACGCAGGGCCTGATGCCA	TGG	TAGGTAACGCAGGGCCTGATGCCA	AAACTGGCATCAGGCCCTGCGTTA
pax3 T2	GGGCTAATTCAGGGCCAGAC	TGG	TAGGGCTAATTCAGGGCCAGAC	AAACGTCTGGCCCTGAATTAGC
pax3 T3	CGGCAGCTCAGGGTTTCCCA	TGG	TAGGCGGCAGCTCAGGGTTTCCCA	AAACTGGGAAACCCTGAGCTGCCG
ctnnb	GGAAAGCAGCAGTCAGTCAC	TGG	TAGGAAAGCAGCAGTCAGTCAC	AAACGTGACTGACTGCTGCTTT
zic1	GATGAGGCTGGGCTTCTCTG	GGG	TAGGATGAGGCTGGGCTTCTCTG	AAACCAGAGAAGCCCAGCCTCAT
ap2a	GGGATACCGCACCAGCTCTC	TGG	TAGGGATACCGCACCAGCTCTC	AAACGAGAGCTGGTGCGGTATC
c-myc	GGAGATGGTGACCGAATTCC	TGG	TAGGAGATGGTGACCGAATTCC	AAACGGAATTCGGTCACCATCT
snail1	GGTGGGCTCGGAGATTGGAG	CGG	TAGGTGGGCTCGGAGATTGGAG	AAACCTCCAATCTCCGAGCCCA
snail1 T2	GGGGGTGGGATTGGGGTGGG	AGG	TAGGGGGTGGGATTGGGGTGGG	AAACCCCACCCCAATCCCACCCCC
snail2	GGAGAGCCCCATCAGCGATG	AGG	TAGGAGAGCCCCATCAGCGATG	AAACCATCGCTGATGGGGCTCT
tyr	GGAACTGGCCCCTGCAAACA	TGG	TAGGAACTGGCCCCTGCAAACA	AAACTGTTTGCAGGGGCCAGTT
foxD3	GGACATCGATGTGGTGGGCG	AGG	TAGGACATCGATGTGGTGGGCG	AAACCGCCCACCACATCGATGT
sox9	GGAGCTGAAGAAGGAGACAG	AGG	TAGGAGCTGAAGAAGGAGACAG	AAACCTGTCTCCTTCTTCAGCT
sox9 T2	TTGGGGAAAGTGTTCTCTTG	GGG	TAGGTTGGGGAAAGTGTTCTCTTG	AAACCAAGAGAACACTTTCCCCAA
ets1	*GGCTGCTCCAGGACTGAGTG*	*AGG*	*TAGGCTGCTCCAGGACTGAGTG*	*AAACCACTCAGTCCTGGAGCAG*
ets1 T2	TTGCCGAGCCATAAATCCAA	AGG	TAGGTTGCCGAGCCATAAATCCAA	AAACTTGGATTTATGGCTCGGCAA
ets2	GGCCGCCAAGGAATTCTCCT	TGG	TAGGCCGCCAAGGAATTCTCCT	AAACAGGAGAATTCCTTGGCGG
id3	GGCAGCAGCCATAAGGGACC	AGG	TAGGCAGCAGCCATAAGGGACC	AAACGGTCCCTTATGGCTGCTG
inca	GGGTTCCTCCAGTCACGACA	AGG	TAGGGTTCCTCCAGTCACGACA	AAACTGTCGTGACTGGAGGAAC
lrig3	TGAAGATGATTCATGGAACT	GGG	TAGGTGAAGATGATTCATGGAACT	AAACAGTTCCATGAATCATCTTCA
lrig3 3'UTR	GGGATTTTTCTATTAAAATA	AGG	TAGGTATTCATCTTGGAATGTACA	AAACTGTACATTCCAAGATGAATA
myoX	GGTTGACAGATGCCAGGATG	**GAG**	TAGGTTGACAGATGCCAGGATG	AAACCATCCTGGCATCTGTCAA
id3	GGCAATGCTTAGACTCTGCT	**CAG**	TAGGCAATGCTTAGACTCTGCT	AAACAGCAGAGTCTAAGCATTG
twist1	GGGATCCTCAGGGCTTTTCC	**TAG**	TAGGGATCCTCAGGGCTTTTCC	AAACGGAAAAGCCCTGAGGATC

Supplementary Table 1

**primers for T7EI assay and colony PCR at the target sites with PAM -NGG**			
**Target gene**	**Primer 1**	**Primer 2**	**Primer 3**
pax3	GCTGACAGGACCCAGGACTAA	CAGGGCCTGATGCCATG	CGCAGGAGAGTGTGAGAGCTT
ctnnb	AATGGAGCTGGACATGGCC	GGAAAGCAGCAGTCAGTCAC	CTGAGAGAAACCTTGCTCCCA
zic1	TGATTTTCCCAGGTCTCCA	GAGGCTGGGCTTCTCTGG	GGCTGCCTCATGTAACGAA
ap-2a	CGCCCTACCAGCCAATAT	CGGGGGATACCGCACCAGC	GTATGGAGTGTAAAGGCAGGT
c-myc	CCAGCAAAGCCGACTGCA	CGAGAAGCCGCTCCACATA	CAGCTGGAGATGGTGACCG
snai1	GAAATTGCCGACTGTCCCC	TGGGCTCGGAGATTGGAG	TGCACCCTGTTTCCTCGT
snai2	AGCGATCTCTCCCCTCTCT	AGTCTTTCCTCCTCATCGCT	TGTGCTTAGCCAACCCAGA
tyr	ATGGTCCCTCTGGCATTCT	CCTCCATGTTTGCAGGGGCC	CATTGGCACGTCCTGTTGTA
foxD3	GCCCTGCCGAGCCTATTTC	ACCACATCGATGTCCGCA	CCGAGTTCGTCCGCTTCCT
sox9	CCAGCCGCTCAACTCTCT	ACCCGGAGCTGAAGAAGGA	GGCTTGCTCTTGCTGGAT
ets1	TTGGTGGGCAGGAATCATT	CCTCACTCAGTCCTGGAGC	GTGTAGCCAGCTAGAGCAGC
ets2	CTGATCCCTTGCAGTGTGG	AATTCACGTTTTCCAAGGAGA	GGAAGACTGGCTCGTCCA
id3	AGCCATCAGCCCAGTGAG	AGGGGCAGCAGCCATAAG	TTCCCCCAGGACTATCTGC
inca	TGGCATTGTGGAGGAGGA	GTGGGTTCCTCCAGTCAC	AGACAGGCTGACAAGGAAGC
lrig3	CAATCTTTACATCCCACTTCC	ACTGTCTTCAATCAATCCCAG	CTGGTACAATTTGCAGTTCAT
lrig3 3'utr	GCTGGATACATAGCCGCATT	GTATATTCATCTTGGAATGTACATG	TGCAAGTGTACAAGGCCTACA
**primers for long fragment deletion and inversion assay**			
pax3 ts1F	ATGTGCTTTTGGTTGGCATT		
pax3 ts1R	CTGTGCGACTTGCAACAACT		
pax3 ts2F	GACTTTCAGGGGTGGATTTG		
pax3 ts2R	TGGGCCTCTGTGTACTAGGG		
snai1 ts1f	GAGCAGAGAGGGCATGTAGG		
snai1 ts1r	GATTTTGGATGGTGCCTCTG		
snai1 ts2f	TTGTGCAAGTCCATTGTGGT		
snai1 ts2r	CTGTTTCCTCGTGCTTGTGA		
**primers for colony PCR at the target sites with PAM -NAG**			
**Target gene**	**Primer 1**	**Primer 2**	**Primer 3**
myoX	GCTGTCATACGCTACTTATCTGCT	GACAGATGCCAGGATGGAG	ATTGTCATGACGCTTCCACA
id3	CCCTGGCACCAACTCTTTTA	AGCCATAAGGGACCAGGTG	AAGCCATCAGCCCAGTGA
twist1	CCCAGTCTCCCCAGTAGACA	GATCCTCAGGGCTTTTCCTAGT	CTGCGAAGGCTTCATTTAGG

Supplementary Table 2

On-target/ Off-target	Reference	Position	Coding/ Non-coding	Sequence
**Irig3 - exon2**	**gi|288985368|ref|NW_003163937.1|**	**608368**	**C**	**TGAAGATGATTCATGGAACTGGG**
Irig3-E306	gi|288974306|ref|NW_003174987.1|	7525	N	TAAAGAGGATTCATGGAAATGGG
Irig3-E306	gi|288974306|ref|NW_003174987.1|	7390	N	TAAAGAGGATTCATGGAAATGGG
**Irig3 - 3'UTR**	**gi|288985368|ref|NW_003163937.1|**	**620127**	**N**	**GGGATTTTTCTATTAAAATAAGG**
Irig3-U931	gi|288985931|ref|NW_003163374.1|	2112551	N	GGCATTTTTCTATTAAAACAGGG
Irig3-U890	gi|288985890|ref|NW_003163415.1|	1856747	N	TGAATTTTTCTATTAAAATGGGG
Irig3-U827	gi|288985827|ref|NW_003163478.1|	1830431	N	GAGATTTGTCTATCAAAATATGG
Irig3-U571	gi|288985571|ref|NW_003163734.1|	752484	N	TAGATTTTTCTATTAGAATAGGG
Irig3-U967	gi|288985967|ref|NW_003163338.1|	6705	N	TGTATTATTCTATTAAAATATGG
Irig3-U967	gi|288985967|ref|NW_003163338.1|	28014	N	TGTATTATTCTATTAAAATATGG
**sox9**	**gi|288985803|ref|NW_003163502.1|**	**1305840**	**C**	**GGAGCTGAAGAAGGAGACAGAGG**
sox9-815	gi|288985815|ref|NW_003163490.1|	629385	N	GGAGGTGAAGAAGGAGACAGGGG
sox9-807	gi|288984807|ref|NW_003164498.1|	90668	N	GCAGCTAAAGAAGGAGACAGAGG
sox9-794a	gi|288985794|ref|NW_003163511.1|	655986	N	GAAGCTGAAGAAGGAGACAGGGG
sox9-794b	gi|288985794|ref|NW_003163511.1|	1172360	N	GAAGCTGAAGAAGGAGACAGGGG

sox9-826	gi|288985826|ref|NW_003163479.1|	329806	N	GGAACTGAATAAGGAGACAGGGG
sox9-766	gi|288985766|ref|NW_003163539.1|	1033563	N	GAAGCTGAAGAAGGATACAGGGG
sox9-600	gi|288985600|ref|NW_003163705.1|	294512	N	GAAGCTGAAGAAGGAAACAGGGG
sox9-863	gi|288985863|ref|NW_003163442.1|	1884871	N	TGAGATGAAGGAGGAGACAGAGG
sox9-579	gi|288985579|ref|NW_003163726.1|	865328	C	AGAGGTGAAAAAGGAGACAGAGG
**foxd3**	**gi|288985975|ref|NW_003163330.1|**	**5300676**	**C**	**GGACATCGATGTGGTGGGCGAGG**
foxd3-745	gi|288985745|ref|NW_003163560.1|	849099	N	GGACAGGGATGTGGTGGGGGGGG
**pax3**	**gi|288985755|ref|NW_003163550.1|**	**892370**	**C**	**TAACGCAGGGCCTGATGCCATGG**
pax3-534	gi|288985534|ref|NW_003163771.1|	524010	C	TAAGGCATGTCCTGATGCCACGG
**zic1**	**gi|288985695|ref|NW_003163610.1|**	**1003587**	**C**	**GATGAGGCTGGGCTTCTCTGGGG**
zic1-871	gi|288985871|ref|NW_003163434.1|	1242807	N	GATCAAGCTGGGGTTCTCTGTGG
zic1-905	gi|288984905|ref|NW_003164400.1|	47502	N	CATTAGGCTGGGCTTGTCTGGGG
zic1-645	gi|288985645|ref|NW_003163660.1|	463191	N	CATGGGTCTGGGCTTCTCTGTGG
**AP2α**	**gi|288985946|ref|NW_003163359.1|**	**1109930**	**C**	**GGGATACCGCACCAGCTCTCTGG**
AP2α-963	gi|288985963|ref|NW_003163342.1|	590383	N	TGGATAGCGCACCAGGTCTCAGG
**inca**	**gi|288985764|ref|NW_003163541.1|**	**1385923**	**C**	**GGGTTCCTCCAGTCACGACAAGG**

inca-409	gi|288985409|ref|NW_003163896.1|	408790	N	GTCTTCCTCCAGTCACCACAAGG
**tyr**	**gi|288985760|ref|NW_003163545.1|**	**1213668**	**C**	**GGAACTGGCCCCTGCAAACATGG**
tyr-813	gi|288985813|ref|NW_003163492.1|	59728	N	GGGACTGGCCCCTGGAAACAAGG
tyr-951	gi|288985951|ref|NW_003163354.1|	442151	N	GGAGATGGCCCCTTCAAACAGGG
**ctnnb**	**gi|288985314|ref|NW_003163991.1|**	**133962**	**C**	**GGAAAGCAGCAGTCAGTCACTGG**
ctnnb-835	gi|288977835|ref|NW_003171470.1|	15844	N	GAAAAGTAGCAGTGAGTCACTGG
**ets1**	**gi|288985863|ref|NW_003163442.1|**	**1450968**	**C**	**GGCTGCTCCAGGACTGAGTGAGG**
ets1-302	gi|288984302|ref|NW_003165003.1|	3417	N	GGCATCTCCAGGGCTGAGTGAGG
**ets2**	**gi|288985577|ref|NW_003163728.1|**	**296233**	**C**	**GGCCGCCAAGGAATTCTCCTTGG**
**c-myc**	**gi|288985584|ref|NW_003163721.1|**	**1036894**	**C**	**GGAGATGGTGACCGAATTCCTGG**
**id3**	**gi|288985873|ref|NW_003163432.1|**	**2578641**	**C**	**GGCAGCAGCCATAAGGGACCAGG**
**snail1**	**gi|288985931|ref|NW_003163374.1|**	**1055144**	**C**	**GGTGGGCTCGGAGATTGGAGCGG**
**snail2**	**gi|288985896|ref|NW_003163409.1|**	**625775**	**C**	**GGAGAGCCCCATCAGCGATGAGG**

Supplementary Table 3

Off-target sites	Forward Primer	Reverse Primer
pax3-534	GTTTGCTATCATTTGCATTGGAAG	CAAGACACTGGCTCTGAATGTGAC
zic1-871	AAGCTGACTGTATTATATCCCTTGAG	GGAGTTCAGTGGTGCCAGATG
zic1-905	TGGAACCATTAAGCAAGTGCC	ACAAATATCAGGCATCAGATACGG
lrig3-E306	GGTCCTTCAGGAAATTAAGGTCC	CGAATTTATGATGAACAGTTGCAG
lrig3-U931	AGTACTCCAGTTCTAGAGCATGCC	AACCACTGCCATTATAAATCTTGC
lrig3-U890	TTCTGGATAACGGGTCCTATGC	CAGCTATTTTGTTGCACAGAATAGG
lrig3-U827	TTACATAACTGGCTACTGCTCATGG	GTTGTGCTTCTTATAGCAGCTGAAC
lrig3-U571	TTATTGGTTGCTATGGGTTACTGC	AACCAACAAAACGTACACCATTTG
lrig3-U967	CACAACAGCAGGTCTTTGACTACC	TGTTCCCAGGAGACCATAGACC
ctnnb-835	TAGCTAAATAGCTGCACATACGCTC	AATCATGTTACTGACTTGTTGCCTC
ets1-302	CCAGTTTTACCTGTACCCTGATCTC	ATCCCATGTGTTTGGACAGCTAC
inca-409	CTGTATGCCCAATGTATGTAACCC	AGAAATTCACTGCTAATCCATAGCC
ap2a-963	AGCACTAAGTATTTGCTGAGTGCTG	TACAGGGATCTTGTTACCATTTGTG
foxD3-745	ATTTCTCTCCAAATTATTCTAGGCC	TATTGCCAATGGATTTGCTACC
tyr-813	CAAAACTTACTCTTGCCAATACCAC	AGCACCTTGATGGCTTTTAATATC
tyr-951	GTCCAATGCTCTTCATATCATTGG	CATCTCATCATGAAACGTAGAGACC
sox9-815	TGCCCTAACTTACTCAGCAGGTAC	GATGCTAGAAAGTAAAGGTGGGC
sox9-807	AGAAAATGCAGGCTTGTAACTTTAC	ATATGAGCTATACATACAGGGCAGC
sox9-794	GGATTTGGAAGAAGATGGCAAC	GAATGTCGGGGGTGTGTTAATC
sox9-826	TTACCCCACACCTTGAGATAAGG	CTCTTCCCCATCCCATCTGAC
sox9-766	ATGTTCCTTGGATTTGGAAGAAG	AAGCTGGTTAAGAAGAAAAATGTTTC
sox9-600	TCCGAATTCAGTGGAAGAGAGAG	TTCTGCAGTTTGCCTAAAAAGAAG
sox9-863	ATTGTCTGCTCAGCAATTTAAAATG	TTCGCTTGGTGCTACTTCTACTG
sox9-579	TGAAACTACCTGAAATAATGTTGGC	CTGTTTGCTATGGGTTGAAGACC

Supplementary Table 4

Supplementary Fig. 1. Sequences of somatic mutations induced by Cas9 and corresponding sgRNAs in *X. tropicalis* G0 embryos. The 2NLS indicates the sgRNAs were injected together with *Cas9-2NLS*. The sequences in bold are the wild type sequences, and the mutated regions are shaded in gray. The letters highlighted in yellow are the target sites, the letters in green are the PAMs. Red dashes indicated deletions (Δ), while low letters in blue represent insertions (+). The numbers in parentheses showed the number of deleted or inserted base pairs, while numbers in square brackets show the frequencies of the mutations in the sequenced samples.

Supplementary Fig. 2. CRISPR/Cas9 mediated mosaic gene knockout G0 *Xenopus*. (A-D) Pigmentary defects (A, B) or paralyzed upper limb (C, D) induced by coinjection of *Cas9* mRNA and *pax3* sgRNA. (E, F) loss of pigments caused by coinjection of *Cas9* mRNA and *tyrosinase* (*tyr*) sgRNA.

Supplementary Fig. 3. NAG is not an effective PAM sequence in *Xenopus* embryos. T7E1 result showing the mutation efficiency induced by sgRNA (with NAG as PAM) and *Cas9* at *twist1, id3* and *myosin X* genes. Genomic sequence of targeted *myosin X* locus is highlighted in yellow, PAM was in green/purple and underlined. PCR fragments of *myosin X* of 3.9% mutation rate were subcloned into the T-vector. However, none of the sequenced colonies harboured mutations at the *myosin X* locus (myoX, myosin X).

Supplementary Fig. 4. **DNA sequences of off-target mutations induced by CRISPR/Cas9.** For all the panels, wild types of off-target sites are shown on the top. sgRNA-DNA interface sequences are highlighted in yellow, while PAMs are in yellow and underlined. Red dashes indicate deletions (-) and lowercase letters in red indicate insertions (+) or substitutions. All the indels are highlighted in gray. The numbers in parentheses show the number of indels in all sequence number, whereas numbers in square brackets show the frequencies of the mutations in all the sequenced samples.

Supplementary Fig. 5. **Double nicking induced genome editing in the loci of *pax3* and *ets1*.** (A, C) sgRNA pairs targeting sites in the loci of *pax3* (A) or *ets1*(C). Letters in blue are the target sites and the PAMs are highlighted in red. The offset indicates

the base pairs between the 5’ ends of two target sites. The 5’ overhang indicates the base pairs between the cutting sites of Cas9 induced by two sgRNAs. (B, D) Sequencing data of the target sites in the loci of *pax3* (B) or *ets1* (D) induced by D10A with double nicking. Red dashes indicate the deletions. Lowercase letters indicate the insertions.

Supplementary Fig. 6. Germline transmission of mutant G0 *Xenopus*. The G0 frogs harboring disrupted genes as indicated were crossed with wild type frog, respectively. The genomic DNA extracted from individual embryos was PCR amplified, and then analyzed by T7E1 assay (A, C, E, G). The positive PCR amplicons were TA-cloned into pMD-18T and followed by sequencing to confirm germ line transmission of mutations (B, D, F, H). Letters in bold indicate wild type sequence, and PAM sequences were underlined. Red dashes indicate deletions. For each heterozygous embryo, 19 clones were selected randomly for sequencing. Red arrows indicated the DNA samples from embryos that harbor mutations. Con- or Con+, PCR products harboring mutations without or with T7E1 treatment.

Supplementary Table 1. The target sites, PAM and oligos of each locus. The DNA sequences with the NGG PAM targeted in this study and oligonucleotides used to subclone the spacer sequences into the sgRNA expression vector. In some cases, extra nucleotides GG were added to the oligo1 to complement the BsaI cloning site on the sgRNA expression vector. NAG PAMs were shown in bold.

Supplementary Table 2. **Primers used in this study for identifying target site mutations in T7E1 assay and colony PCR.** All of the primer sequences are written in 5'- 3', from left to right.

Supplementary Table 3. **The potential off-target sites of the 16 loci knocked out in this study.** The sequences in bold fond is the target sites. The PAMs are underlined. The mismatched nucleotides in off-target sites are highlighted in red.

Supplementary Table 4. Primers used in this study for identifying off-target effect by T7 Endonuclase 1 and colony PCR in T7E1 and colony PCR. All of the primer sequences are written in 5'- 3', from left to right.
